# Ethyl 2-(4-chloro-2-oxo-2,3-dihydro-1,3-benzothia­zol-3-yl)acetate

**DOI:** 10.1107/S1600536809007727

**Published:** 2009-03-06

**Authors:** Wen-Tong Shen, Cheng Yao

**Affiliations:** a College of Science, Nanjing University of Technolgy, Xinmofan Road No. 5 Nanjing, Nanjing 210009, People’s Republic of China

## Abstract

In the mol­ecule of the title compound, C_11_H_10_ClNO_3_S, the benzene and thia­zole rings are oriented at a dihedral angle of 1.25 (3)°. Intra­molecular C—H⋯O and C—H⋯Cl inter­actions result in the formation of two five-membered rings which both adopt envelope conformations.

## Related literature

For a related structure, see: Shao *et al.* (2001 ▶). For bond-length data, see: Allen *et al.* (1987 ▶).
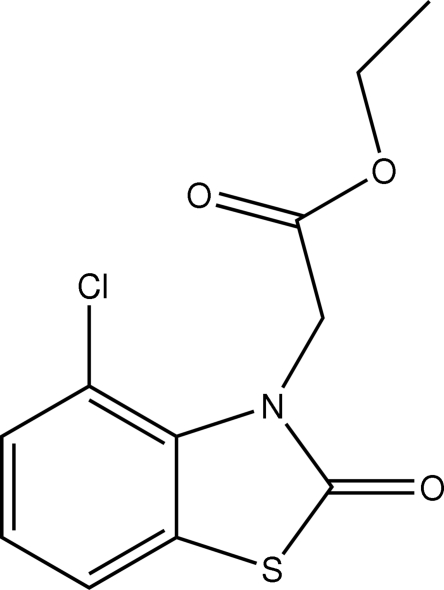


## Experimental

### 

#### Crystal data

C_11_H_10_ClNO_3_S
*M* *_r_* = 271.71
Monoclinic, 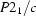

*a* = 5.4830 (11) Å
*b* = 19.410 (4) Å
*c* = 11.060 (2) Å
β = 95.16 (3)°
*V* = 1172.3 (4) Å^3^
*Z* = 4
Mo *K*α radiation
μ = 0.50 mm^−1^
*T* = 294 K
0.20 × 0.10 × 0.10 mm

#### Data collection

Enraf–Nonius CAD-4 diffractometer
Absorption correction: ψ scan (North *et al.*, 1968 ▶) *T*_min_ = 0.907, *T*_max_ = 0.952
2363 measured reflections
2132 independent reflections
1460 reflections with *I* > 2σ(*I*)
*R*_int_ = 0.044
3 standard reflections frequency: 120 min intensity decay: 1%

#### Refinement

*R*[*F* ^2^ > 2σ(*F* ^2^)] = 0.057
*wR*(*F* ^2^) = 0.172
*S* = 1.00
2132 reflections
154 parameters
H-atom parameters constrained
Δρ_max_ = 0.34 e Å^−3^
Δρ_min_ = −0.28 e Å^−3^

### 

Data collection: *CAD-4 Software* (Enraf–Nonius, 1989 ▶); cell refinement: *CAD-4 Software*; data reduction: *XCAD4* (Harms & Wocadlo, 1995 ▶); program(s) used to solve structure: *SHELXS97* (Sheldrick, 2008 ▶); program(s) used to refine structure: *SHELXL97* (Sheldrick, 2008 ▶); molecular graphics: *ORTEP-3 for Windows* (Farrugia, 1997 ▶) and *PLATON* (Spek, 2009 ▶); software used to prepare material for publication: *SHELXTL* (Sheldrick, 2008 ▶).

## Supplementary Material

Crystal structure: contains datablocks global, I. DOI: 10.1107/S1600536809007727/hk2637sup1.cif

Structure factors: contains datablocks I. DOI: 10.1107/S1600536809007727/hk2637Isup2.hkl

Additional supplementary materials:  crystallographic information; 3D view; checkCIF report

## Figures and Tables

**Table 1:** Hydrogen-bond geometry (Å, °)

*D*—H⋯*A*	*D*—H	H⋯*A*	*D*⋯*A*	*D*—H⋯*A*
C4—H4*A*⋯O3	0.97	2.36	2.768 (5)	105
C4—H4*B*⋯Cl	0.97	2.63	3.105 (4)	110

**Fig. 1.**
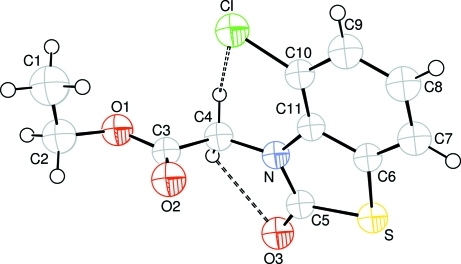
The molecular structure of the title molecule, with the atom-numbering scheme. Hydrogen bonds are shown as dashed lines.

## supplementary crystallographic information

### Comment

The title compound is widely used in preventing cole from pest and is also useful to kill broad-leaved weed. It is likely to be decomposed in the soil. We report herein the crystal structure of the title compound.

In the molecule of the title compound (Fig. 1), the bond lengths (Allen *et al.*,  1987) and angles are within normal ranges. Rings A (C6-C11) and B (S/N/C5/C6/C11) are, of course, planar, and they are oriented at a dihedral angle 1.25 (3)°. So, they are also coplanar. The intramolecular C-H···O and C-H···Cl interactions (Table 1) result in the formations of two five-membered rings C (O3/N/C4/C5/H4A) and D (Cl/N/C4/C10/C11/H4B), adopting envelope conformations with H4A and H4B atoms displaced by -0.284 (3) and -0.661 (3) Å from the planes of the other ring atoms, respectively.

### Experimental

For the preparation of the title compound, 4-chlorobenzothiazol-2(3*H*)-one (10.7 g, 57.5 mmol), ethyl choroacetate (4.3 g, 50 mmol), and the catalyst of potassium iodide (0.63 g, 3 mmol) were added to butyl acetate solution (200 ml) of potassium carbonate (2.72 g, 20 ml) as acid-binding at 353 K. It was stirred for 8 h, and then cooled to room temperature. Water (150 ml) was added to dissolve the product, and inorganic salts were generated. The separated aqueous phase was extracted three times by butyl acetate, and then combined with organic phase product, treated with vacuum distillation at 353 K. Some anhydrous ethanol (about 40 ml) was added to the residual products, the combination was heated into homogeneous phase. Thereafter, precipitated products were cooled (Shao *et al.*, 2001). Crystals suitable for X-ray analysis were obtained by evaporating the solvent slowly at room temperature for about 15 d.

### Refinement

H atoms were positioned geometrically, with C-H = 0.93, 0.97 and 0.96 Å for aromatic, methylene and methyl H, respectively, and constrained to ride on their parent atoms, with U_iso_(H) = xU_eq_(C), where x = 1.5 for methyl H and x = 1.2 for all other H atoms.

### Crystal data

**Table:**

C_11_H_10_ClNO_3_S	*F*(000) = 560
*M**_r_* = 271.71	*D*_x_ = 1.539 Mg m^−^^3^
Monoclinic, *P*2_1_/*c*	Mo *K*α radiation, λ = 0.71073 Å
Hall symbol: -P 2ybc	Cell parameters from 25 reflections
*a* = 5.4830 (11) Å	θ = 10–12°
*b* = 19.410 (4) Å	µ = 0.50 mm^−^^1^
*c* = 11.060 (2) Å	*T* = 294 K
β = 95.16 (3)°	Block, colorless
*V* = 1172.3 (4) Å^3^	0.20 × 0.10 × 0.10 mm
*Z* = 4	

### Data collection

**Table:**

Enraf–Nonius CAD-4 diffractometer	1460 reflections with *I* > 2σ(*I*)
Radiation source: fine-focus sealed tube	*R*_int_ = 0.044
graphite	θ_max_ = 25.3°, θ_min_ = 2.1°
ω/2θ scans	*h* = 0→6
Absorption correction: ψ scan (North *et al.*, 1968)	*k* = 0→23
*T*_min_ = 0.907, *T*_max_ = 0.952	*l* = −13→13
2363 measured reflections	3 standard reflections every 120 min
2132 independent reflections	intensity decay: 1%

### Refinement

**Table:**

Refinement on *F*^2^	Primary atom site location: structure-invariant direct methods
Least-squares matrix: full	Secondary atom site location: difference Fourier map
*R*[*F*^2^ > 2σ(*F*^2^)] = 0.057	Hydrogen site location: inferred from neighbouring sites
*wR*(*F*^2^) = 0.172	H-atom parameters constrained
*S* = 1.00	*w* = 1/[σ^2^(*F*_o_^2^) + (0.1*P*)^2^ + 0.14*P*] where *P* = (*F*_o_^2^ + 2*F*_c_^2^)/3
2132 reflections	(Δ/σ)_max_ < 0.001
154 parameters	Δρ_max_ = 0.34 e Å^−^^3^
0 restraints	Δρ_min_ = −0.28 e Å^−^^3^

### Special details

**Table:**

Geometry. All e.s.d.'s (except the e.s.d. in the dihedral angle between two l.s. planes) are estimated using the full covariance matrix. The cell e.s.d.'s are taken into account individually in the estimation of e.s.d.'s in distances, angles and torsion angles; correlations between e.s.d.'s in cell parameters are only used when they are defined by crystal symmetry. An approximate (isotropic) treatment of cell e.s.d.'s is used for estimating e.s.d.'s involving l.s. planes.
Refinement. Refinement of *F*^2^ against ALL reflections. The weighted *R*-factor *wR* and goodness of fit *S* are based on *F*^2^, conventional *R*-factors *R* are based on *F*, with *F* set to zero for negative *F*^2^. The threshold expression of *F*^2^ > σ(*F*^2^) is used only for calculating *R*-factors(gt) *etc*. and is not relevant to the choice of reflections for refinement. *R*-factors based on *F*^2^ are statistically about twice as large as those based on *F*, and *R*- factors based on ALL data will be even larger.

### Fractional atomic coordinates and isotropic or equivalent isotropic displacement parameters (Å^2^)

**Table:**

	*x*	*y*	*z*	*U*_iso_*/*U*_eq_	
Cl	−0.37482 (19)	0.59357 (6)	0.35838 (10)	0.0538 (4)	
S	0.41735 (19)	0.71164 (6)	0.58220 (10)	0.0472 (3)	
O1	−0.1295 (5)	0.67435 (15)	0.0880 (3)	0.0510 (8)	
O2	0.1424 (5)	0.61068 (17)	0.2030 (3)	0.0568 (8)	
O3	0.3832 (6)	0.78020 (17)	0.3753 (3)	0.0574 (8)	
N	0.0918 (5)	0.69736 (17)	0.4015 (3)	0.0394 (8)	
C1	−0.2905 (10)	0.5683 (3)	−0.0001 (5)	0.0751 (16)	
H1A	−0.2854	0.5377	−0.0681	0.113*	
H1B	−0.4543	0.5853	0.0030	0.113*	
H1C	−0.2413	0.5438	0.0735	0.113*	
C2	−0.1232 (10)	0.6264 (2)	−0.0132 (4)	0.0595 (12)	
H2A	0.0422	0.6091	−0.0163	0.071*	
H2B	−0.1699	0.6502	−0.0888	0.071*	
C3	0.0067 (7)	0.6585 (2)	0.1892 (4)	0.0410 (9)	
C4	−0.0388 (7)	0.7124 (2)	0.2852 (3)	0.0443 (10)	
H4A	0.0119	0.7572	0.2577	0.053*	
H4B	−0.2128	0.7146	0.2945	0.053*	
C5	0.2972 (7)	0.7356 (2)	0.4359 (4)	0.0434 (10)	
C6	0.1986 (7)	0.6473 (2)	0.5897 (3)	0.0400 (9)	
C7	0.1815 (9)	0.6014 (2)	0.6847 (4)	0.0522 (11)	
H7A	0.2950	0.6021	0.7525	0.063*	
C8	−0.0084 (9)	0.5547 (2)	0.6758 (4)	0.0542 (11)	
H8A	−0.0245	0.5236	0.7386	0.065*	
C9	−0.1741 (9)	0.5536 (2)	0.5752 (4)	0.0541 (11)	
H9A	−0.3022	0.5221	0.5708	0.065*	
C10	−0.1530 (7)	0.5992 (2)	0.4797 (4)	0.0419 (9)	
C11	0.0334 (7)	0.64719 (19)	0.4852 (3)	0.0384 (9)	

### Atomic displacement parameters (Å^2^)

**Table:**

	*U*^11^	*U*^22^	*U*^33^	*U*^12^	*U*^13^	*U*^23^
Cl	0.0421 (6)	0.0549 (7)	0.0633 (7)	−0.0090 (5)	−0.0018 (5)	−0.0008 (5)
S	0.0446 (6)	0.0507 (7)	0.0456 (6)	−0.0038 (5)	−0.0001 (5)	−0.0043 (5)
O1	0.0571 (18)	0.0488 (18)	0.0446 (16)	0.0022 (14)	−0.0087 (14)	−0.0003 (14)
O2	0.0544 (18)	0.059 (2)	0.0563 (19)	0.0132 (16)	−0.0005 (15)	−0.0047 (15)
O3	0.0624 (19)	0.056 (2)	0.0527 (18)	−0.0162 (16)	0.0011 (15)	0.0084 (15)
N	0.0380 (17)	0.0366 (18)	0.0427 (18)	−0.0012 (14)	−0.0014 (14)	0.0015 (15)
C1	0.081 (4)	0.069 (4)	0.076 (4)	−0.017 (3)	0.005 (3)	−0.010 (3)
C2	0.075 (3)	0.059 (3)	0.044 (2)	−0.009 (3)	0.000 (2)	−0.007 (2)
C3	0.034 (2)	0.043 (2)	0.046 (2)	−0.0049 (18)	−0.0014 (17)	0.0029 (19)
C4	0.045 (2)	0.042 (2)	0.045 (2)	0.0046 (19)	−0.0004 (18)	0.0060 (19)
C5	0.042 (2)	0.045 (2)	0.043 (2)	0.0010 (19)	0.0037 (18)	−0.0022 (19)
C6	0.041 (2)	0.038 (2)	0.042 (2)	0.0050 (18)	0.0049 (17)	−0.0026 (17)
C7	0.067 (3)	0.046 (3)	0.043 (2)	0.005 (2)	0.007 (2)	0.005 (2)
C8	0.066 (3)	0.041 (3)	0.056 (3)	−0.001 (2)	0.011 (2)	0.009 (2)
C9	0.065 (3)	0.040 (2)	0.059 (3)	−0.009 (2)	0.018 (2)	0.001 (2)
C10	0.0351 (19)	0.040 (2)	0.050 (2)	0.0047 (18)	0.0011 (17)	−0.0030 (19)
C11	0.041 (2)	0.032 (2)	0.043 (2)	0.0057 (17)	0.0079 (17)	−0.0037 (17)

### Geometric parameters (Å, °)

**Table:**

S—C6	1.738 (4)	C2—H2A	0.9700
S—C5	1.755 (4)	C2—H2B	0.9700
Cl—C10	1.731 (4)	C3—C4	1.527 (6)
O1—C3	1.325 (5)	C4—H4A	0.9700
O1—C2	1.459 (5)	C4—H4B	0.9700
O2—C3	1.191 (5)	C6—C7	1.388 (6)
O3—C5	1.215 (5)	C6—C11	1.403 (5)
N—C5	1.373 (5)	C7—C8	1.377 (6)
N—C11	1.400 (5)	C7—H7A	0.9300
N—C4	1.445 (5)	C8—C9	1.372 (7)
C1—C2	1.469 (7)	C8—H8A	0.9300
C1—H1A	0.9600	C9—C10	1.390 (6)
C1—H1B	0.9600	C9—H9A	0.9300
C1—H1C	0.9600	C10—C11	1.381 (5)
			
C6—S—C5	91.77 (19)	C3—C4—H4B	109.1
C3—O1—C2	116.7 (3)	H4A—C4—H4B	107.9
C5—N—C11	115.1 (3)	O3—C5—N	125.6 (4)
C5—N—C4	117.7 (3)	O3—C5—S	124.4 (3)
C11—N—C4	127.2 (3)	N—C5—S	110.0 (3)
C2—C1—H1A	109.5	C7—C6—C11	122.7 (4)
C2—C1—H1B	109.5	C7—C6—S	126.3 (3)
H1A—C1—H1B	109.5	C11—C6—S	111.0 (3)
C2—C1—H1C	109.5	C8—C7—C6	118.1 (4)
H1A—C1—H1C	109.5	C8—C7—H7A	121.0
H1B—C1—H1C	109.5	C6—C7—H7A	121.0
O1—C2—C1	110.9 (4)	C9—C8—C7	120.6 (4)
O1—C2—H2A	109.5	C9—C8—H8A	119.7
C1—C2—H2A	109.5	C7—C8—H8A	119.7
O1—C2—H2B	109.5	C8—C9—C10	120.8 (4)
C1—C2—H2B	109.5	C8—C9—H9A	119.6
H2A—C2—H2B	108.1	C10—C9—H9A	119.6
O2—C3—O1	126.0 (4)	C11—C10—C9	120.5 (4)
O2—C3—C4	125.7 (4)	C11—C10—Cl	122.8 (3)
O1—C3—C4	108.3 (3)	C9—C10—Cl	116.6 (3)
N—C4—C3	112.4 (3)	C10—C11—N	130.8 (4)
N—C4—H4A	109.1	C10—C11—C6	117.3 (4)
C3—C4—H4A	109.1	N—C11—C6	112.0 (3)
N—C4—H4B	109.1		
			
C3—O1—C2—C1	82.2 (5)	C6—C7—C8—C9	0.5 (7)
C2—O1—C3—O2	4.0 (6)	C7—C8—C9—C10	0.5 (7)
C2—O1—C3—C4	−176.4 (3)	C8—C9—C10—C11	−1.1 (7)
C5—N—C4—C3	104.5 (4)	C8—C9—C10—Cl	−179.3 (4)
C11—N—C4—C3	−75.3 (5)	C9—C10—C11—N	179.7 (4)
O2—C3—C4—N	−3.7 (6)	Cl—C10—C11—N	−2.2 (6)
O1—C3—C4—N	176.7 (3)	C9—C10—C11—C6	0.5 (6)
C11—N—C5—O3	177.8 (4)	Cl—C10—C11—C6	178.7 (3)
C4—N—C5—O3	−2.0 (6)	C5—N—C11—C10	−178.2 (4)
C11—N—C5—S	−3.0 (4)	C4—N—C11—C10	1.6 (7)
C4—N—C5—S	177.2 (3)	C5—N—C11—C6	0.9 (5)
C6—S—C5—O3	−177.5 (4)	C4—N—C11—C6	−179.3 (3)
C6—S—C5—N	3.3 (3)	C7—C6—C11—C10	0.5 (6)
C5—S—C6—C7	177.6 (4)	S—C6—C11—C10	−179.1 (3)
C5—S—C6—C11	−2.8 (3)	C7—C6—C11—N	−178.8 (4)
C11—C6—C7—C8	−1.0 (6)	S—C6—C11—N	1.6 (4)
S—C6—C7—C8	178.5 (3)		

### Hydrogen-bond geometry (Å, °)

**Table:**

*D*—H···*A*	*D*—H	H···*A*	*D*···*A*	*D*—H···*A*
C4—H4A···O3	0.97	2.36	2.768 (5)	105
C4—H4B···Cl	0.97	2.63	3.105 (4)	110
